# Respiratory Induced Modulation in f-Wave Characteristics During Atrial Fibrillation

**DOI:** 10.3389/fphys.2021.653492

**Published:** 2021-04-08

**Authors:** Mostafa Abdollahpur, Fredrik Holmqvist, Pyotr G. Platonov, Frida Sandberg

**Affiliations:** ^1^Department of Biomedical Engineering, Lund University, Lund, Sweden; ^2^Department of Cardiology, Clinical Sciences, Lund University, Lund, Sweden

**Keywords:** atrial fibrillation, autonomic nervous system, ECG processing, f-wave frequency, parasympathetic regulation, respiratory modulation

## Abstract

The autonomic nervous system (ANS) is an important factor in cardiac arrhythmia, and information about ANS activity during atrial fibrillation (AF) may contribute to personalized treatment. In this study we aim to quantify respiratory modulation in the f-wave frequency trend from resting ECG. First, an f-wave signal is extracted from the ECG by QRST cancelation. Second, an f-wave model is fitted to the f-wave signal to obtain a high resolution f-wave frequency trend and an index for signal quality control (S). Third, respiratory modulation in the f-wave frequency trend is extracted by applying a narrow band-pass filter. The center frequency of the band-pass filter is determined by the respiration rate. Respiration rate is estimated from a surrogate respiration signal, obtained from the ECG using homomorphic filtering. Peak conditioned spectral averaging, where spectra of sufficient quality from different leads are averaged, is employed to obtain a robust estimate of the respiration rate. The envelope of the filtered f-wave frequency trend is used to quantify the magnitude of respiratory induced f-wave frequency modulation. The proposed methodology is evaluated using simulated f-wave signals obtained using a sinusoidal harmonic model. Results from simulated signals show that the magnitude of the respiratory modulation is accurately estimated, quantified by an error below 0.01 Hz, if the signal quality is sufficient (S>0.5). The proposed method was applied to analyze ECG data from eight pacemaker patients with permanent AF recorded at baseline, during controlled respiration, and during controlled respiration after injection of atropine, respectively. The magnitude of the respiratory induce f-wave frequency modulation was 0.15 ± 0.01, 0.18 ± 0.02, and 0.17 ± 0.03 Hz during baseline, controlled respiration, and post-atropine, respectively. Our results suggest that parasympathetic regulation affects the magnitude of respiratory induced f-wave frequency modulation.

## 1. Introduction

Despite progress in atrial fibrillation (AF) treatment, such as ablation procedures, stroke-prevention procedures, and anti-arrhythmic drugs, AF still is associated with significant mortality in middle-aged and older adults, and it constitutes a substantial burden to the health economy (Hindricks et al., 2020). The current estimate of AF prevalence for adults in the United States is ranged between 2 and 4% (Benjamin et al., 2019). The prevalence of AF increases with age and is higher in men. In a Swedish study including 30,447 individuals, AF prevalence was 1.5% higher in men and increased from 2 per 1,000 in ages 45–49 to 29 per 1,000 in ages 70–74 (Smith et al., 2010). There are some substantial modifiers which contribute to the maintenance and progression of AF, such as atrial fibrosis and aging, ion-channel dysfunction, autonomic imbalance, and genetic background (Fabritz et al., 2016). Better understanding and monitoring of these AF-causing factors can contribute to personalized AF treatment.

Autonomic dysfunction is one of the main factors which can contribute to AF (Fabritz et al., 2016). The autonomic nervous system (ANS) plays an important role in cardiac arrhythmogenesis. Previous research has established an understanding of the cardiac ANS and provided evidence to support the relationship between autonomic tone and cardiac arrhythmia (Shen and Zipes, 2014). For example, low-level vagal stimulation has been shown to suppress AF episodes in ambulatory dogs (Shen et al., 2011). Further, experimental studies has shown that changes in sympathetic or parasympathetic tone may change the atrial action potential and refractory period (Liu and Nattel, 1997; Sharifov et al., 2004).

The atrial electrical activity during AF can be characterized from the f-waves in the ECG; f-wave amplitude, f-wave frequency, f-wave morphology, f-wave regularity, and f-wave complexity has been proposed for this purpose (Petrutiu et al., 2006; Meo et al., 2013; Lankveld et al., 2014; Sörnmo, 2018). Such f-wave characteristics has been suggested for prediction of treatment outcome, e.g., a low f-wave amplitudes predicted AF recurrence after catheter ablation in a study including 54 patients with persistent AF (Cheng et al., 2013), and large f-wave amplitude predicted termination of AF during catheter ablation in another study including 90 patients with persistent AF (Nault et al., 2009). Also, Lankveld et al. (2016) found the chances of successful in cather ablation in patients with persistent AF can be predicted by AF complexity and frequency parameters; the study included 91 patients for training of the prediction models and validated by 83 patients.

The f-wave frequency, often referred to as the atrial fibrillatory rate, has received considerable clinical attention (Platonov et al., 2014). Low f-wave frequency can predict successful outcome in patient with persistent AF undergoing cardioversion (Bollmann et al., 2008) and high f-wave frequency predicts early AF recurrence (Bollmann et al., 2003). The f-wave frequency can increase with the progression of AF, and patients with persistent AF often have a higher f-wave frequency than patients with paroxysmal AF (Alcaraz et al., 2011; Park et al., 2019). Further, it has been shown that a low f-wave frequency is associated with spontaneous conversion of recent-onset AF (Choudhary et al., 2013). However, the link between f-wave frequency and progression of disease is ambiguous since a low f-wave frequency is also associated with poor outcome in heart failure patients with long-standing AF (Platonov et al., 2012).

Previous studies have shown that the f-wave frequency can change in response to changes in autonomic tone. The f-wave frequency has been shown to increase in response to head-up tilt (Ingemansson et al., 1998; Östenson et al., 2017) and decrease in response to head-down tilt (Östenson et al., 2017). Further, the f-wave frequency has been shown to follow a circadian pattern where it increases during daytime and decreases at night (Meurling et al., 2001; Sandberg et al., 2010). Controlled respiration can induce cyclic fluctuations in the f-wave frequency. Holmqvist et al. (2005) found that the spectral power of the f-wave frequency trend in the respiratory frequency band increased in response to controlled respiration and decreased in response to vagal blockade for eight patients with permanent AF. However, individual variations were large. In another study using a similar methodology, the f-wave frequency was influenced by controlled respiration and attenuated by the vagal blockade in only two out of eight patients with permanent AF (Stridh et al., 2003).

The aim of this study is to develop a methodology that can be used to quantify respiratory induced variations in the f-wave frequency from resting ECG. This is challenging, since (1) respiratory induced f-wave frequency modulation is very small and may be concealed by other variations and (2) the respiration rate is unknown and may vary over time. A preliminary version of this work, where the respiration rate was assumed to be known, was presented at the CinC conference 2020 (Abdollahpur et al., 2020). In contrast, the respiration rate in the present study is estimated from the ECG.

## 2. Materials and Methods

**Figure 1:**
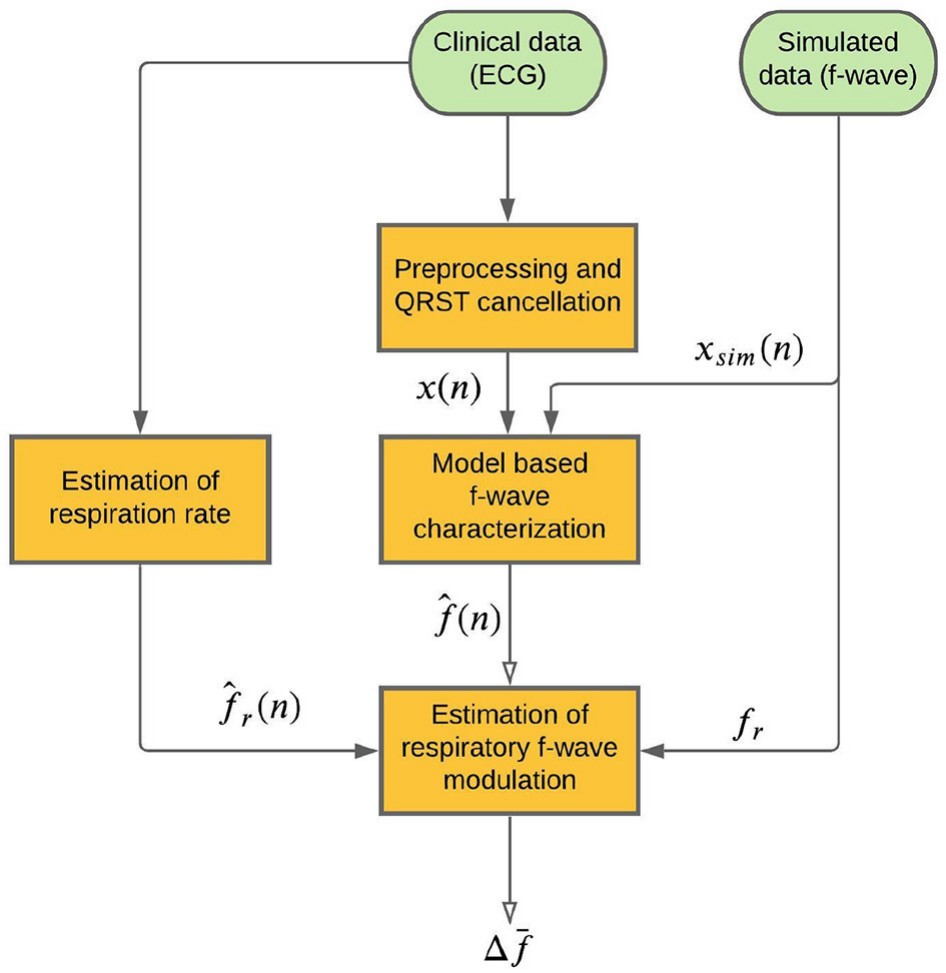
Schematic of the proposed method.

A schematic outline of the methodology is shown in Figure 1. An f-wave signal *x*(*n*) is extracted from the ECG by QRST cancelation (section 2.3). A model-based approach to f-wave characterization is applied to estimates an f-wave frequency trend $\hat{f}\left(n\right)$ and a signal quality index S from *x*(*n*) (section 2.4). Respiratory modulation in $\hat{f}\left(n\right)$ is estimated using a bandpass filtering approach (section 2.5). A respiration rate estimate ${\hat{f}}_{r}\left(n\right)$, which is required for the bandpass filtering, is derived from the ECG (section 2.6). The accuracy of the estimated respiratory modulation magnitude $\Delta \bar{f}$ is evaluated using simulated f-wave signals *x*_*sim*_(*n*) (section 2.1). Finally, the method is applied to analyse data from a clinical study (section 2.2).

### 2.1. Simulated Data

A modified version of the saw-tooth model (Stridh and Sörnmo, 2001) is used to simulate f-wave signals. The simulated f-waves consists of the sum of a sinusoid with time-varying frequency and its harmonic

(1) $$\begin{array}{l} x_{sim}\left(n\right)=\overset{2}{\underset{k=1}{\sum }}a_{k}\left(n\right)\sin\left(2\pi kf\left(n\right)n\right)+v\left(n\right) \end{array}$$

The time-varying frequency is given by

(2) $$\begin{array}{l} f\left(n\right)=\frac{f}{f_{s}}+\frac{\Delta f}{2\pi f_{r}n}\sin\left(2\pi n\frac{f_{r}}{f_{s}}\right)+\frac{\Phi \left(n\right)}{2\pi kn} \end{array}$$

where *f* defines the average fundamental frequency, and respiratory f-wave frequency modulation is quantified by *f*_*r*_ and Δ*f*, defining the modulation frequency and the modulation magnitude, respectively. Random phase variation, Φ(*n*), is added to account for other variations in the f-wave frequency; it is modeled as white Gaussian noise with standard deviation σ_Φ_. The amplitude of the *k*th harmonic is given by

(3) $$\begin{array}{l} a_{k}\left(n\right)=\frac{2}{k\pi }\left(a+\Delta a\left(n\right)\right) \end{array}$$

where *a* is the average f-wave amplitude, and Δ*a*(*n*) quantifies random amplitude variation and is assumed to have a Gaussian distribution with mean zero and standard deviation *a*/5.

**Figure 2:**
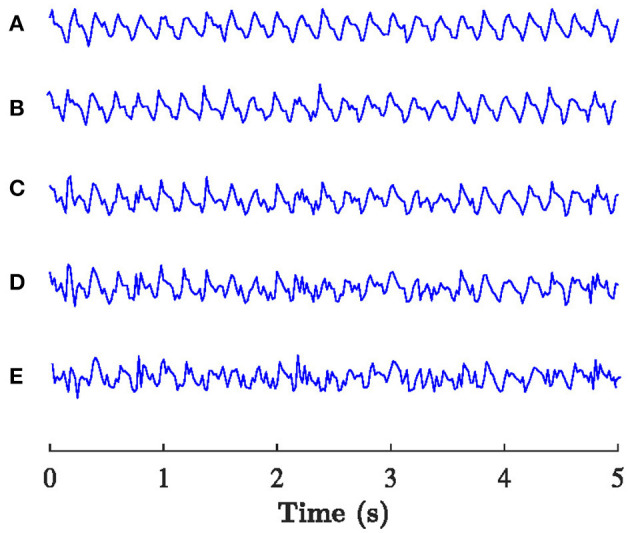
Example of the *x*_*sim*_(*n*) with *f* = 5 Hz, *f*_*r*_ = 0.2 Hz, Δ*f* = 0.1 Hz, and **(A)** σ_Φ_= 0, **(B)** σ_Φ_= 0.25, **(C)** σ_Φ_= 0.5, **(D)** σ_Φ_= 0.75, **(E)** σ_Φ_=1.

The following parameters are used for the simulations f-wave signals: *f* = {5, 6, 7} Hz, *f*_*r*_ = {0.1, 0.2, 0.3} Hz, Δ*f* = {0, 0.1, 0.2} Hz, *a* chosen such that standard deviation of signal σ_*x*_ = 50 μV, and σ_Φ_ = {0, 0.25, 0.5, 0.75, 1}. White Gaussian noise *v*(*n*) with standard deviation of 0.1*a* is added to form realistic f-wave signals and the sampling frequency was set to *f*_*s*_ = 50*Hz*. For each parameter setting 10 realizations of *x*_*sim*_(*n*) were considered, resulting in a total of 1,350 simulated signals. Examples of *x*_*sim*_(*n*) with different values of σ_Φ_ are displayed in Figure 2.

### 2.2. Clinical Data

The study population consists of eight pacemaker patients with permanent AF and complete atrioventricular block that participated in a clinical study (Holmqvist et al., 2005). The clinical characteristics of the study population is summarized in Table 1. The study was conducted in accordance with the Declaration of Helsinki, and the protocol was approved by the local Ethics Committee. All subjects gave their informed consent for inclusion before they participated in the study. The study protocol consisted of three phases; baseline rest (B), controlled respiration (CR), and controlled respiration following injection of atropine to induce full vagal blockade (PA), respectively. Each phase lasted 5 min, and standard 12-lead ECG at 1 kHz sampling rate was recorded throughout the study protocol. During controlled respiration, the patients inhaled for 4 s and exhaled for 4 s, following instructions from the study nurse.

**Table 1:** Patient characteristics.

**Patient**	**Age, yr**	**Gender**	**EF,%**	**Left atrial diameter (mm)**	**AF duration, mo**	**Number of cardioversions**	**Heart active drugs**	**Comorbidity**
a	62	Female	55	41	7	10	Diltiazem	None
b	64	Male	45	43	5	15	None	COPD
c	65	Female	35	37	9	4	Metoprolol	HT, CHF
d	64	Female	55	32	30	4	None	None
e	54	Male	55	46	24	7	Losartan	None
f	59	Male	55	53	32	10	Losartan	HT
g	68	Female	55	45	7	26	Spironolactone	None
h	53	Male	55	32	24	7	Enalapril	HT
Gross average	61 ± 5		51 ± 7	41 ± 7	17 ± 11	10 ± 7		

*CHF, chronic heart failure; COPD, chronic obstructive pulmonary disease; EF, ejection fraction; HT, hypertension*.

### 2.3. Preprocessing and QRST Cancelation

Following preprocessing, atrial activity was extracted from ventricular activity in the ECG using a spatiotemporal QRST cancelation technique (Stridh and Sörnmo, 2001). Briefly, a scaled, spatial, and temporally aligned average QRS complexes is subtracted from each QRS complex in the ECG, the Cardiolund ECG Parser was used for this task. Since the resulting f-wave signal has negligible frequency content above 25 Hz, it was down-sampled to 50 Hz. In the present study, the extracted f-wave signal from lead V1 was subjected to analysis; this signal is denoted *x*(*n*).

### 2.4. Model Based f-Wave Characterization

The harmonic f-wave model (Henriksson et al., 2018) is employed to estimate the local f-wave frequency, phase, and amplitude. In this model, f-waves are formulated by the complex signal *s*(*n*; θ), defined as the sum of a complex exponential signal with fundamental frequency *f* and its second harmonic,

(4) $$\begin{array}{l} s\left(n;\theta \right)=\overset{2}{\underset{m=1}{\sum }}\;A_{m}e^{j\left(m2\pi \frac{f}{f_{s}}n+\phi_{m}\right)} \end{array}$$

where *A*_*m*_ and ϕ_*m*_ define the amplitude and phase, respectively, of the *m*:th harmonic, and *f*_*s*_ is sampling frequency of *x*(*n*).

The model is fitted to the complex-valued analytic representation of *x*(*n*), denoted *x*_*a*_(*n*), and obtained using Hilbert transformation. The parameter vector $\theta ={\left[f\;A_{1}\;A_{2}\;\phi_{1}\;\phi_{2}\right]}^{T}$ is estimated using a maximum likelihood approach

(5) $$\begin{array}{l} \hat{\theta }=\underset{\theta }{\arg\min}{\left\|x_{a}\left(n\right)-s\left(n;\theta \right)\right\|}^{2} \end{array}$$

The model is fitted to 20 ms overlapping 0.5 s segments of *x*_*a*_(*n*); the local estimates $\hat{f}$ results in an f-wave frequency trend $\hat{f}\left(n\right)$ sampled at 50 Hz.

In the present study, we analyse 5 min recordings obtained during stable conditions and therefore we assume that the f-wave frequency will not change drastically within the recording. Hence, the local estimation of $\hat{f}$ is constrained to the interval $\left[{\hat{f}}_{0}-1.5,{\hat{f}}_{0}+1.5\right]$ Hz, where ${\hat{f}}_{0}$ is an initial f-wave frequency estimate determined by the maximum spectral peak in the interval [4, 12] Hz of the Welch periodogram of the entire recording *x*(*n*).

The model error $ê\left(n\right)=x_{a}\left(n\right)-s\left(n;\hat{\theta }\right)$ is used to estimate a signal quality index,

(6) $$\begin{array}{l} S=1-\frac{\sigma_{\hat{e}}}{\sigma_{x_{a}}} \end{array}$$

where σ_ê_ and σ_*x*_*a*__ denote the standard deviation of ê(*n*) and *x*_*a*_(*n*), respectively (Henriksson et al., 2018). For any reasonable estimate of $s\left(n;\hat{\theta }\right)$, S is limited to the interval [0, 1], where one represents the best model fit. A poor model fit, quantified by a low value of S suggests that the parameter estimates $\hat{\theta }$ are unreliable. In the present study, S is estimated based on the entire 5-min recording.

### 2.5. Estimation of Respiratory f-Wave Modulation

A forth-order Butterworth band pass filter with a fixed bandwidth β (Raja Kumar and Pal, 1985) and a center frequency determined by the respiration rate ${\hat{f}}_{r}\left(n\right)$ is employed to extract respiratory modulation in $\hat{f}\left(n\right)$. The bandwidth of filter is set to 0.06 Hz since the magnitude of respiration rate estimation error is expected to be constrained to this range (Kontaxis et al., 2020). The transfer function of the filter is given by

(7) $$\begin{array}{l} H\left(z\right)=\frac{a_{0}+a_{2}z^{-2}+a_{4}z^{-4}}{1+b_{1}Wz^{-1}+\left(b_{2}W^{2}+b_{2}\prime \right)z^{-2}+b_{3}Wz^{-3}+b_{4}z^{-4}} \end{array}$$

where the coefficients are given by

(8) $$\begin{array}{l} \begin{array}{l} a_{0}=1/\left(k^{2}+\sqrt{2}k+1\right)\\ a_{2}=-2a_{0}\\ a_{4}=a_{0}\\ b_{1}=-2k\left(2k+\sqrt{2}\right)a_{0}\\ b_{2}=4k^{2}a_{0}\\ b_{2}\prime =2\left(k^{2}-1\right)a_{0}\\ b_{3}=2k\left(-2k+\sqrt{2}\right)a_{0}\\ b_{4}=\left(k^{2}-\sqrt{2}k+1\right)a_{0} \end{array} \end{array}$$

and *k* and *W* are given by

(9) $$\begin{array}{l} \begin{array}{l} k=cot\left(\pi \beta /f_{s}\right)\\ W=\frac{cos\left(2\pi f_{r}/f_{s}\right)}{cos\left(\pi \beta /f_{s}\right)} \end{array} \end{array}$$

respectively. The output of the filter is denoted $\tilde{f}\left(n\right)$. An estimate of the magnitude of the respiratory f-wave frequency modulation is given by the envelope of $\tilde{f}\left(n\right)$, obtained as the magnitude of its analytic equivalent using Hilbert transformation. The estimate is denoted $\Delta \hat{f}\left(n\right)$. Since $\Delta \hat{f}\left(n\right)$ varies over time, we use its 5 min average $\Delta \bar{f}$ to quantify the magnitude of respiratory f-wave frequency modulation in this study.

### 2.6. Estimation of Respiration Rate

**Figure 3:**
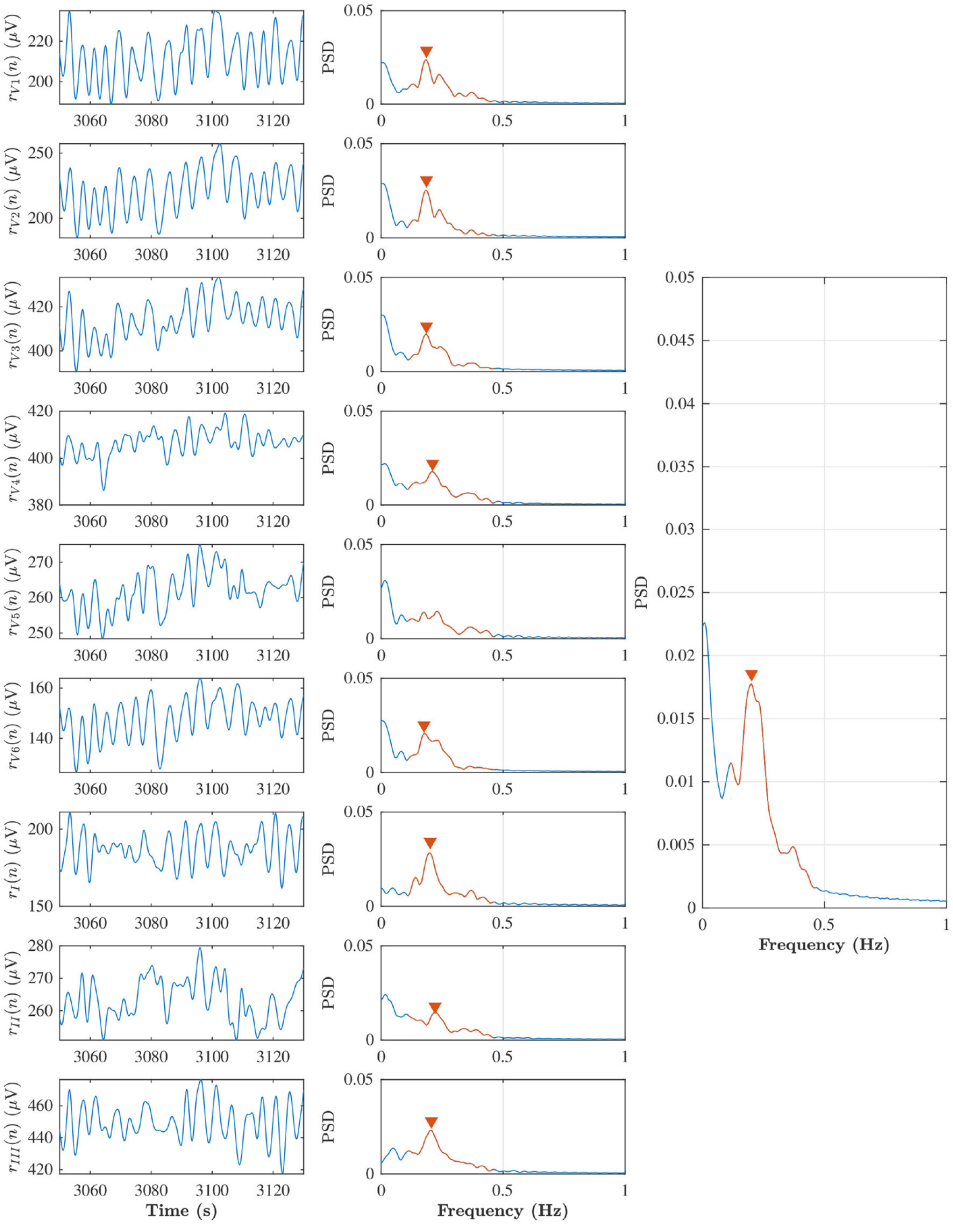
Left subplots indicate *r*_*l*_(*n*) from lead V1, V2, V3, V4, V5, V6, I, II, and III during baseline in the patient (b), and middle subplots are corresponding Welch periodogram. The prominent peak in the respiratory interval (red solid line) is shown with red marker. The averaged spectra and respiration rate estimate can be seen in the right subplot.

A surrogate respiration signal is derived from the ECG leads (V1, V2, V3, V4, V5, V6, I, II, III) by using homomorphic filtering to extract slow variations in the amplitudes (Rezek and Roberts, 1998). First, the ECG signal is decimated to 50 Hz and a zero-phase first-order Butterworth low-pass filter with a cut-off frequency of 2 Hz is applied to emphasize slow variation in the ECG amplitude caused by chest movements during the respiratory cycle. Then, the peak envelope of the filtered ECG signal is determined and smoothed using a Savitzky-Golay filter with polynomial order four and frame length 251 corresponding to 5 s, and the resulting envelope is down-sampled to 5 Hz. A similar approach is applied separately to each ECG lead; the surrogate respiration signal obtained from lead *l* is denoted *r*_*l*_(*n*). A robust estimate of the respiration rate is obtained by combining *r*_*l*_(*n*) obtained from all ECG leads using peak conditioned spectral averaging (Bailón et al., 2006; Lázaro et al., 2013; Sandberg et al., 2019). In this technique, Welch periodograms are estimated from sliding segments of *r*_*l*_(*n*) from each lead, and periodograms of sufficient quality are averaged to produce a power spectrum from which the respiration rate can be estimated more robustly. In the present study, Welch periodograms were computed based on 80 s sliding 75 s overlapping segments of *r*_*l*_(*n*), by averaging power spectra of 50% overlapping 24 s subintervals. A periodogram is considered to be of sufficient quality if it has a prominent peak in the respiratory interval, defined by at least 85% of the maximal peak height in the spectrum. The respiration rate estimates obtained from the averaged spectra every 5 s are denoted ${\hat{f}}_{r}\left(n\right)$. An example of *r*_*l*_(*n*), corresponding Welch periodograms, and averaged power spectra obtained from an 80 s time segment from patient b in phase B, is displayed in Figure 3. In this example, the average spectrum used for respiration rate estimation is based on all leads except lead V5, for which the periodogram was considered of insufficient quality.

## 3. Results

The estimation accuracy of $\Delta \bar{f}$ is evaluated using simulations and the estimation accuracy of ${\hat{f}}_{r}\left(n\right)$ is evaluated using the CR and PA phases of the clinical data for which the respiration rate is known. Finally, results from analysis of clinical data during B, CR, and PA are presented.

### 3.1. Estimation Accuracy of Respiratory f-Wave Modulation

**Figure 4:**
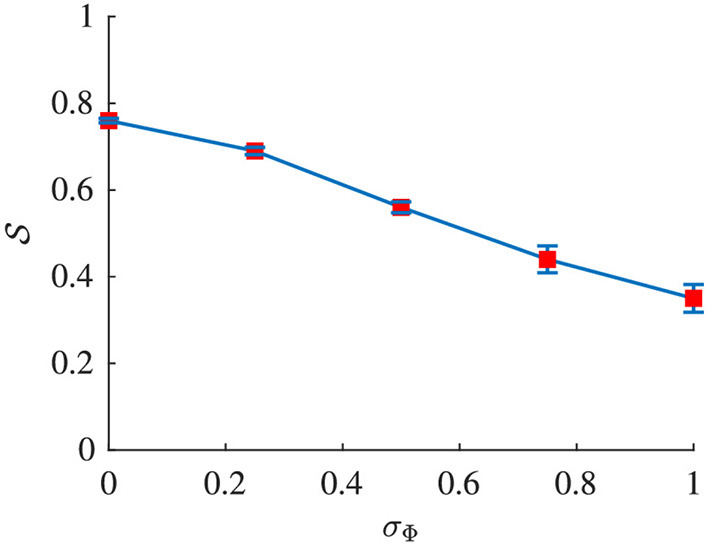
Signal quality S of *x*_*sim*_(*n*) plotted vs. σ_Φ_. Red dots indicate the mean and blue whiskers indicate the std of S.

**Figure 5:**
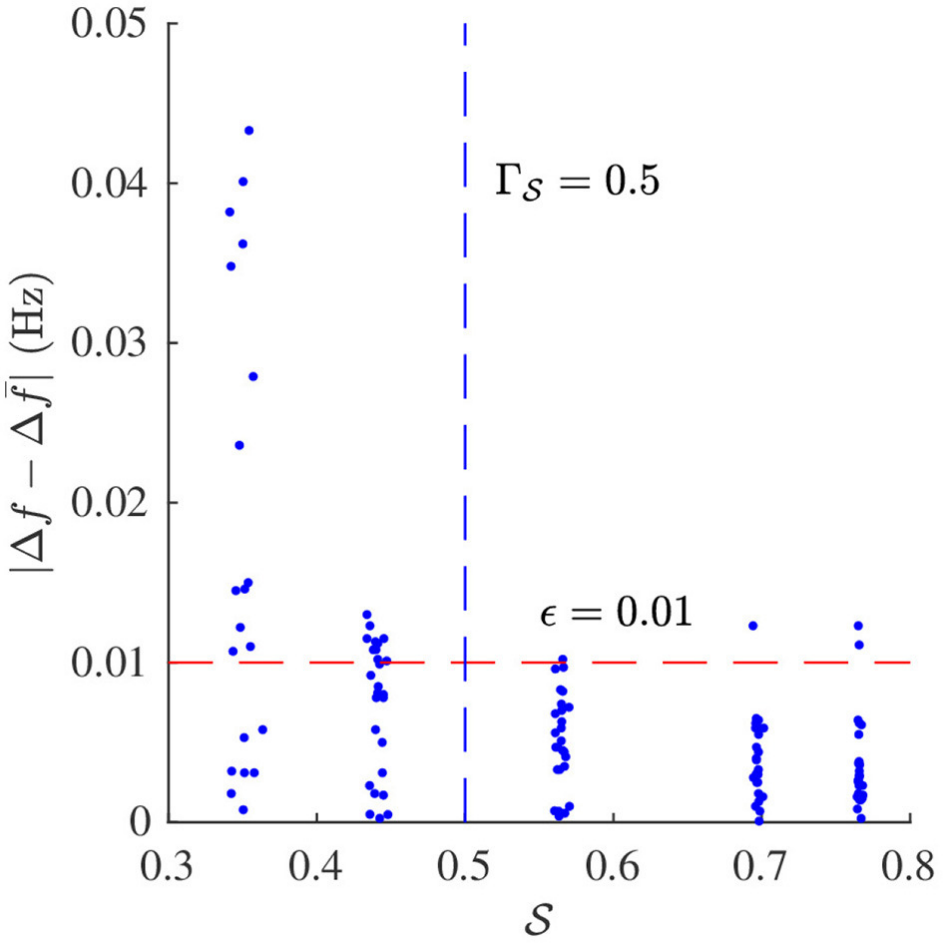
Estimation error $|\Delta \bar{f}$-Δ*f*| and corresponding signal quality S of *x*_*sim*_(*n*).

Signal quality S and $\Delta \bar{f}$ were estimated from the simulated f-wave signals using the methods described in sections 2.4 and 2.5, respectively. The sampling frequency *f*_*s*_ in Equation (4) was set to 50 Hz to match the sampling frequency of the simulated f-wave signals. The respiration rate used for the band-pass filtering was set to *f*_*r*_ as used for the corresponding simulation. Results from the analysis of simulated data are presented in Figures 4, 5. Figure 4 shows that S decreases with increasing σ_Φ_ independently of the other parameter settings. The estimation error, quantified by the absolute difference between Δ*f* and $\Delta \bar{f}$ is displayed in Figure 5. Results suggest that Δ*f* can be accurately estimated if S is above $\Gamma_{S}=0.5$; 95% of the estimates has an error below 0.01 Hz if S>0.5. For S<0.5 the estimation error becomes large which indicates that the estimate $\Delta \bar{f}$ is unreliable. Hence a threshold of $\Gamma_{S}=0.5$ was used to determine if the signal quality is sufficient for analysis.

### 3.2. Estimation of Respiration Rate in Clinical Data

Respiration rate ${\hat{f}}_{r}\left(n\right)$ was estimated from the clinical dataset using the method described in section 2.6. Table 2 summarizes the estimated respiration rates ${\hat{f}}_{r}\left(n\right)$ for each patient during B, CR, and PA, respectively. The standard deviation of ${\hat{f}}_{r}\left(n\right)$ within each recording is smaller than the bandwidth of the filter β, implying that the center frequency *f*_*r*_ can be fixed to the mean ${\bar{f}}_{r}$.

**Table 2:** Estimated respiration rate ${\hat{f}}_{r}\left(n\right)$ (mean ± std) for B, CR, and P, respectively.

**Patient**	**${\hat{f}}_{r_{B}}$ (*Hz*)**	**${\hat{f}}_{r_{CR}}$ (*Hz*)**	**${\hat{f}}_{r_{PA}}$ (*Hz*)**
a	0.268 ± 0.007	0.125 ± 0.000	0.125 ± 0.000
			
b	0.252 ± 0.006	0.162 ± 0.000^*^	0.180 ± 0.056^*^
c	0.208 ± 0.008	0.125 ± 0.000	0.125 ± 0.000
			
d	0.215 ± 0.007	0.125 ± 0.000	0.125 ± 0.001
			
e	0.232 ± 0.003	0.125 ± 0.000	0.125 ± 0.000
			
f	0.308 ± 0.002	0.125 ± 0.000	0.125 ± 0.000
			
g	0.232 ± 0.019	0.125 ± 0.000	0.125 ± 0.000
			
h	0.163 ± 0.003	0.125 ± 0.000	0.125 ± 0.000
Gross average	0.235 ± 0.043	0.129 ± 0.013	0.131 ± 0.019

* *Indicates that ${\hat{f}}_{r}\left(n\right)$ differed significantly from the true respiration rate*.

The estimation accuracy of ${\hat{f}}_{r}\left(n\right)$ was evaluated on the CR and PA phases, for which the respiration rate is known to be 0.125 Hz. In all patients except one, ${\hat{f}}_{r}\left(n\right)$ gave an accurate estimate of the true respiration rate in the CR and PA phases (see Table 2). It should be noted that patient b, for which ${\hat{f}}_{r}\left(n\right)$ did not correspond to 0.125 Hz in CR and PA, has a considerable amount of ectopic beats, which may explain why the respiration rate estimation failed. This patient was excluded from further analysis.

### 3.3. Estimation of Respiratory f-Wave Modulation in Clinical Data

**Figure 6:**
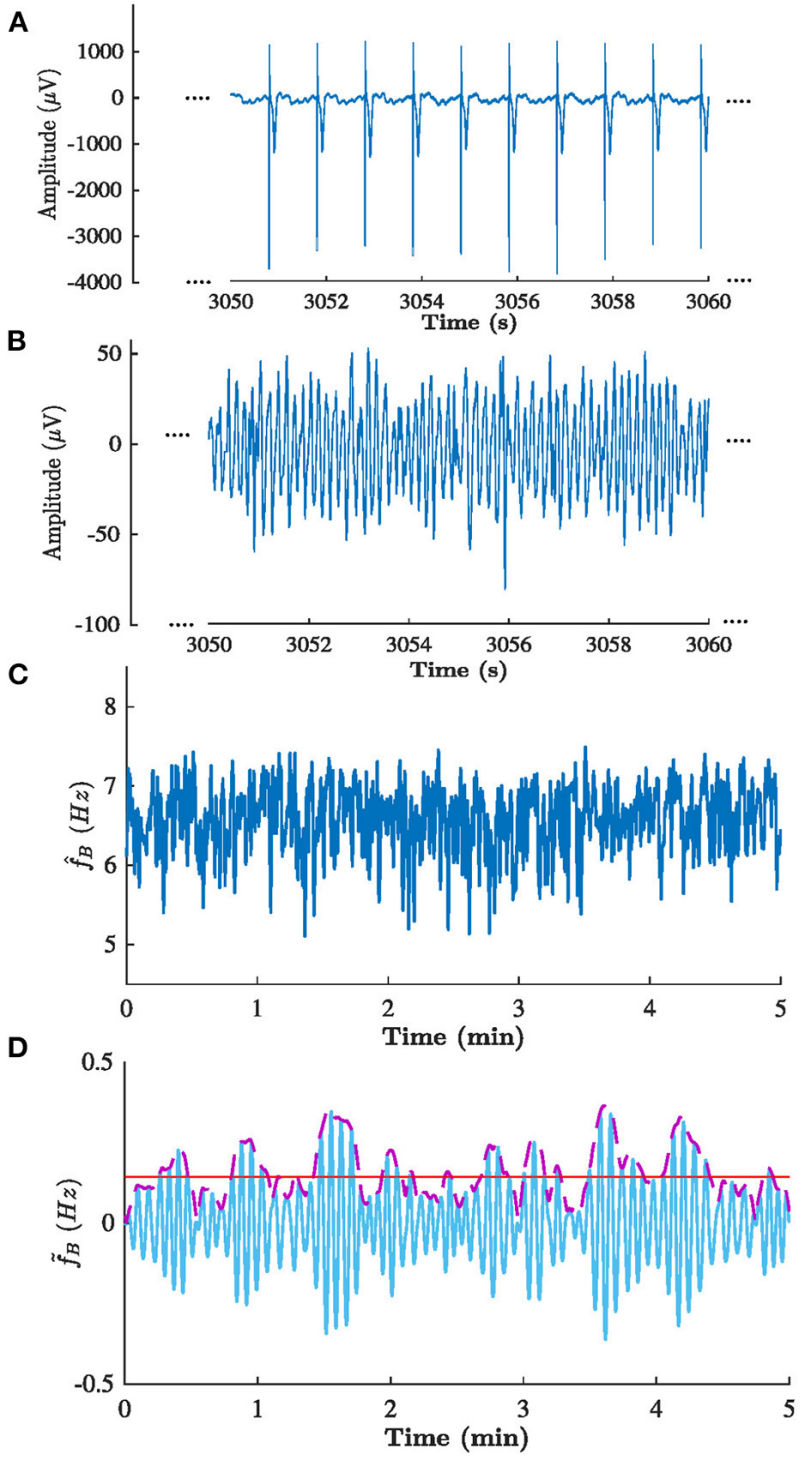
Signals obtained in each step of the analysis of patient b in phase B. **(A)** ECG from lead V1, **(B)** corresponding extracted f-waves *x*(*n*), **(C)** estimated f-wave frequency trend $\hat{f}\left(n\right)$, and **(D)** corresponding (solid blue) filtered $\hat{f}\left(n\right)$, (dashed purple) $\Delta \hat{f}\left(n\right)$, and (solid red) estimated $\Delta \bar{f}$. Note the that **(A,B)** shows 10 s excerpts of the signals, whereas **(C,D)** shows the full 5 min signal.

The methodology as described in sections 2.3–2.6 was applied to analyze the clinical data described in section 2.2. The sampling frequency *f*_*s*_ in Equation (4). was set to 50 Hz to match the sampling frequency of the f-wave signals. Figure 6 illustrates the signals obtained in each step of the analysis for patient b in phase B.

**Figure 7:**
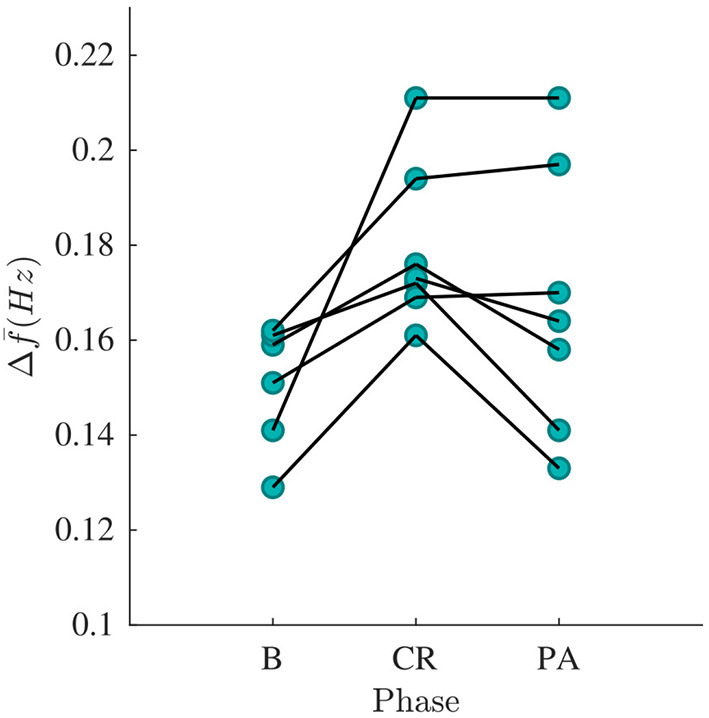
$\Delta \bar{f}$ estimated from phase B, CR, and PA recordings, respectively. Each curve corresponds to a patient.

The signal quality was sufficient for analysis ($S>\Gamma_{S}$) in all recordings except one. The mean and standard deviation of $\hat{f}\left(n\right)$ and $\Delta \hat{f}\left(n\right)$ are shown in the Table 3. A Kruskal-Wallis test with Dunn-Sidak correction was applied to analyze if the differences between phases for each patient were significant. Results indicate that $\Delta \hat{f}\left(n\right)$ was significantly larger in CR than in B for all patients, and that $\Delta \hat{f}\left(n\right)$ was significantly smaller in PA than in CR for four patients (*p* < 0.05). For $\hat{f}\left(n\right)$ the results were more heterogeneous; $\hat{f}\left(n\right)$ was significantly larger in CR than in B for three patients and significantly smaller in CR than in B for three patients (*p* < 0.05). Further, $\hat{f}\left(n\right)$ was significantly larger in PA than in CR for four patients and significantly smaller in PA than in CR for three patients (*p* < 0.05). The gross average $\Delta \hat{f}$ was 0.15 ± 0.01 Hz (mean±std) during B, 0.18 ± 0.02 Hz during CR, 0.17 ± 0.03 Hz during PA. There is a trend toward increased $\Delta \bar{f}$ during CR and decreased $\Delta \bar{f}$ during PA (see Figure 7). A Friedman test was applied to analyze if the differences in $\Delta \bar{f}$ between B, CR, and PA were significant. Results indicate that only the changes between B and CR are significant.

**Table 3:** Estimated f-wave frequency $\hat{f}\left(n\right)$ and respiratory frequency modulation $\Delta \hat{f}\left(n\right)$ (mean ± std) from clinical data.

**Patient**	**$\Delta {\hat{f}}_{B}\left(n\right)\left(Hz\right)$**	**$\Delta {\hat{f}}_{CR}\left(n\right)\left(Hz\right)$**	**$\Delta {\hat{f}}_{PA}\left(n\right)\left(Hz\right)$**	**${\hat{f}}_{B}\left(n\right)\left(Hz\right)$**	**${\hat{f}}_{CR}\left(n\right)\left(Hz\right)$**	**${\hat{f}}_{PA}\left(n\right)\left(Hz\right)$**
a	0.16 ± 0.06	0.17 ± 0.09^*^	0.16 ± 0.08^•^	6.84 ± 0.43	6.77 ± 0.45^*^	6.71 ± 0.41^•*^
c	0.13 ± 0.06	0.16 ± 0.08^*^	0.13 ± 0.07^•^	6.17 ± 0.35	6.31 ± 0.37^*^	6.46 ± 0.36^•*^
d	0.16 ± 0.08	0.17 ± 0.08^*^	0.14 ± 0.07^•*^	6.57 ± 0.43	6.75 ± 0.39^*^	6.36 ± 0.43^•*^
fe	0.16 ± 0.08	0.19 ± 0.10^*^	0.19 ± 0.10^*^	7.68 ± 0.51	7.39 ± 0.54^*^	7.57 ± 0.54^•*^
f	0.14 ± 0.07	0.21 ± 0.10^*^	0.21 ± 0.12^*^	7.46 ± 0.46	7.29 ± 0.49^*^	7.14 ± 0.48^•*^
g	—	0.17 ± 0.08	0.16 ± 0.08^•^	—	5.91 ± 0.41	5.96 ± 0.42^•^
h	0.15 ± 0.08	0.17 ± 0.07^*^	0.17 ± 0.07^*^	7.73 ± 0.43	8.11 ± 0.44^*^	7.98 ± 0.43^•*^
Gross average	0.15 ± 0.01	0.18 ± 0.02	0.17 ± 0.03	7.07 ± 0.64	6.93 ± 0.73	6.88 ± 0.71

Subscripts {B, CR, PA} indicates phase of recording, (^*^) indicates significant differences to B and (^•^) indicates significant differences to CR. The phase B recording from patient g was excluded from analysis due insufficient signal quality ($S<\Gamma_{S}$).

**Figure 8:**
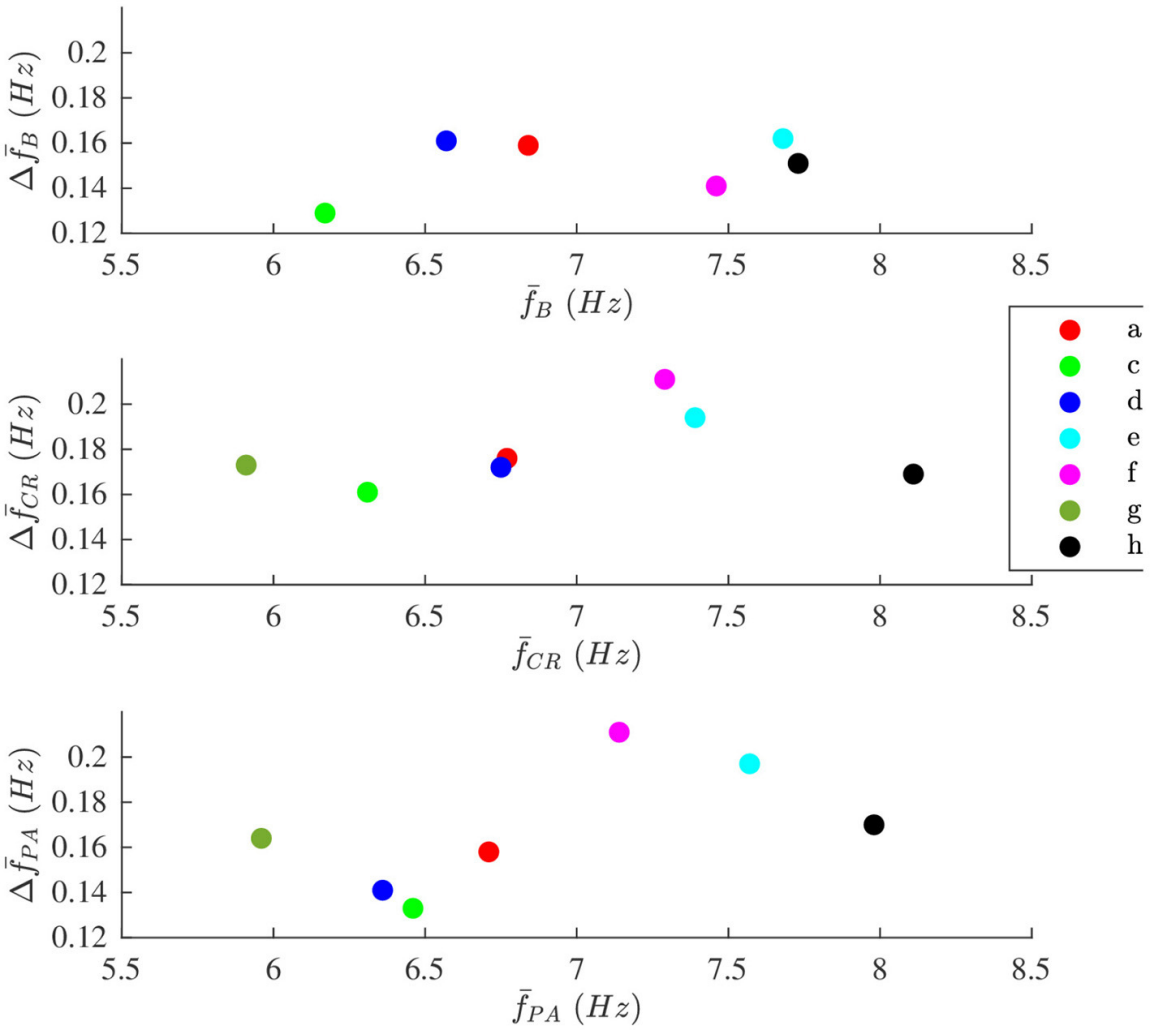
The modulation magnitude $\Delta \bar{f}$ plotted vs. average the f-wave frequency $\bar{f}$ for each patient during **(top)** B, **(middle)** CR, and **(bottom)** PA phase, respectively. Patients are identified with different colors.

The modulation magnitude $\Delta \bar{f}$ is plotted vs. the average $\hat{f}\left(n\right)$, denoted $\bar{f}$, in Figure 8. There is no correlation between $\Delta \bar{f}$ and $\bar{f}$ in any of the phases.

## 4. Discussion

The aim of the study was to develop a method to quantify respiratory modulation in the f-wave frequency. Simulation results shows that the method works accurately provided that the signal quality is sufficient (cf. Figure 5). Our results from analysis of clinical data suggest that the magnitude of the respiratory f-wave frequency modulation provide complementary information to the average f-wave frequency (cf. Figure 8).

In previous studies that have investigated respiratory modulation in the f-wave frequency, a spectral approach was used (Stridh et al., 2003; Holmqvist et al., 2005). In this study, we use a recently proposed model-based approach that allows more detailed f-wave characterization and provides a signal quality metric S that can be used to exclude unreliable frequency estimates caused by artifacts in the f-wave signal (Henriksson et al., 2018). It should be noted that the settings in the present study were different from the ones used in Henriksson et al. (2018), to facilitate analysis of respiratory modulation in f-wave frequency trend. Since the recordings in the present study were obtained during stationary conditions, the initial frequency estimate was based on the entire 5 min recording rather than on 5 s segments of the recording. Further, we allowed a larger local frequency deviation from the initial estimate; 1.5 Hz rather than 0.25 Hz. In Henriksson et al. (2018), it was shown that S larger than 0.3 was sufficient for accurate estimation of $\hat{f}\left(n\right)$. Our simulation results indicate that a similar S is required for accurate estimation of the average f-wave frequency with the present settings. Analysis of small variations in the frequency trend, however, requires better signal quality, and our simulation results indicate that S larger than 0.5 is required for accurate estimation of respiratory f-wave frequency modulation.

The proposed methodology relies on ECG derived respiration rate estimation. It should be mentioned such estimation requires ECG length sufficiently long due to respiratory frequency during baseline phase. If the respiration was known the method could be apply to shorter segment. Several methods have been proposed to extract respiratory information from the ECG. One of the most common approaches is to use respiratory sinus arrhythmia, i.e., respiratory induced variations in the heart rate (Charlton et al., 2018). However, such approach is not feasible during AF where the heart beats result from complex interactions between the atria and the atrioventricular node. Another approach is to use beat-to-beat morphological variations in the QRS complexes caused by chest movements, using e.g., vectorcardiogram loop analysis (Bailón et al., 2006). Such analysis is more challenging in AF due to presence of f-waves, however, a recent study showed that respiration rate could be accurately estimated from the ECG using a method based on the differences between the maximal upslope and the minimal downslope within a QRS interval (Kontaxis et al., 2020). In the present study we analyze ECG recordings from patients with pacemakers. The pacemaker causes sharp spikes in the signal and, hence, the previously proposed methods based on morphological variations in the QRS complex are not applicable. Instead, we exploit variation in the ECG amplitude caused by chest movements to estimate the respiration rate. Our results show that the estimated respiration rate corresponded to the expected respiration rate during controlled respiration in all patients except one (cf. Table 3). For that patient we found that a considerable amount of ectopic beats caused the respiration rate estimation to fail.

In the present study we used a filtering approach to extract respiratory variations in the f-wave frequency trend. Adaptation of the filter to varying respiration rates is possible, however, in the present data the respiration rate was found to be constant within each phase and no adaptation of the filter was required. Another approach to extract respiratory variations in the f-wave frequency trend would be to use orthogonal subspace projections. In this approach the f-wave frequency trend can be decomposed into two different components, one respiratory component, and one residual component by a projection matrix. Such approach has previously been used to remove respiratory influence in the heart rate for improved heart rate variability analysis (Varon et al., 2019). In contrast to the filtering approach which relies on the respiration rate, the subspace projections approach requires that a respiratory signal is available.

It has been shown that respiratory modulation in heat rate, i.e., respiratory sinus arrhythmia, can be used for non-invasive assessment of parasympathetic activity (Katona and Jih, 1975; Alcalay et al., 1992). In this study we aim to quantify respiratory modulation in the atrial activity during AF. Our results from clinical data shows that the magnitude of respiratory f-wave frequency modulation increase with deep breathing (increased parasympathetic activity) and decrease with vagal block (decreased parasympathetic tone), suggesting that respiratory modulation in the f-wave frequency trend can be partly attributed to parasympathetic regulation in the atria during AF. This is supported by a recent simulation study that showed that the parasympathetic neurotransmitter acetylcholine could be an important factor involved in f-wave frequency modulation (Celotto et al., 2020). After injection of atropine, there is still considerable variation in the respiration frequency band; these variations may be caused by other factors such as the endocrine system (Gordan et al., 2015) or stretch of the atrial tissue induced by respiratory chest movements.

### 4.1. Limitations

The proposed methodology requires ECG recordings longer than the 10 s clinical standard. The requirement that is motivated by the respiratory cycle length, which is assumed to be between 10 and 2.5 s corresponding to a respiration rate of 6–24 breaths per minute. Therefore, a 10 s ECG segment may contain only one complete respiratory cycle which is insufficient for robust analysis of respiratory modulation. The methodology was tested in a small group of AF patients with pacemakers in controlled settings and the feasibility of the methodology has to be verified in a larger study population. Further, the clinical significance of respiratory induced f-wave modulation remains to be established.

## 5. Conclusions

We introduce a novel approach to quantify respiratory induced variations in the f-wave frequency from the ECG. Results from simulated signals indicate that respiratory modulation can be accurately estimated when the signal quality is sufficient. Results from analysis of clinical data suggest that respiratory f-wave frequency modulation increase during deep breathing and decrease after injection of atropine, implying that parasympathetic ANS regulation is a contributing factor to the modulation.

## Data Availability Statement

The raw data supporting the conclusions of this article will be made available by the authors, without undue reservation.

## Ethics Statement

The studies involving human participants were reviewed and approved by the local ethic committee and complied with the Declaration of Helsinki. The patients/participants provided their written informed consent to participate in this study.

## Author Contributions

FS devised the project and the main conceptual ideas and was responsible for overseeing the research and providing critical insight and recommendations regarding the focus, structure, and content of the paper. MA processed the data, performed the analysis, drafted the manuscript, and designed the figures. FH was responsible for the clinical data acquisition protocol. PP helped supervise the project focusing on clinical aspects. All authors contributed to the final version of the manuscript.

## Conflict of Interest

The authors declare that the research was conducted in the absence of any commercial or financial relationships that could be construed as a potential conflict of interest.
