# Supplementary material for: Respiratory Induced Modulation in f-Wave Characteristics During Atrial Fibrillation
Source: Front Physiol. 2021 Apr 8;12:653492. doi: 10.3389/fphys.2021.653492 (PMC8060635; doi:10.3389/fphys.2021.653492)
Supplement: Supplementary file 1 [file Data_Sheet_1.PDF]

frontiers

Supplementary Material: Respiratory Induced Modulation in f-wave Characteristics During Atrial Fibrillation

1 SUPPLEMENTARY TABLES AND FIGURES

Table S1: Parameters settings for simulated signals.

$f(Hz)$	$f_r(Hz)$	$\Delta f(Hz)$	σ_Φ	Number of realization
5	0.1	0	0	10
5	0.1	0	0.25	10
5	0.1	0	0.5	10
5	0.1	0	0.75	10
5	0.1	0	1	10
5	0.1	0.1	0	10
5	0.1	0.1	0.25	10
5	0.1	0.1	0.5	10
5	0.1	0.1	0.75	10
5	0.1	0.1	1	10
5	0.1	0.2	0	10
5	0.1	0.2	0.25	10
5	0.1	0.2	0.5	10
5	0.1	0.2	0.75	10
5	0.1	0.2	1	10
5	0.2	0	0	10
5	0.2	0	0.25	10
5	0.2	0	0.5	10
5	0.2	0	0.75	10
5	0.2	0	1	10
5	0.2	0.1	0	10
5	0.2	0.1	0.25	10
5	0.2	0.1	0.5	10
5	0.2	0.1	0.75	10
5	0.2	0.1	1	10
5	0.2	0.2	0	10
5	0.2	0.2	0.25	10
5	0.2	0.2	0.5	10
5	0.2	0.2	0.75	10
5	0.2	0.2	1	10
5	0.3	0	0	10
5	0.3	0	0.25	10

Continued on next page

Table S1 – continued from previous page

$f(Hz)$	$f_r(Hz)$	$\Delta f(Hz)$	σ_Φ	Number of realization
5	0.3	0	0.5	10
5	0.3	0	0.75	10
5	0.3	0	1	10
5	0.3	0.1	0	10
5	0.3	0.1	0.25	10
5	0.3	0.1	0.5	10
5	0.3	0.1	0.75	10
5	0.3	0.1	1	10
5	0.3	0.2	0	10
5	0.3	0.2	0.25	10
5	0.3	0.2	0.5	10
5	0.3	0.2	0.75	10
5	0.3	0.2	1	10
6	0.1	0	0	10
6	0.1	0	0.25	10
6	0.1	0	0.5	10
6	0.1	0	0.75	10
6	0.1	0	1	10
6	0.1	0.1	0	10
6	0.1	0.1	0.25	10
6	0.1	0.1	0.5	10
6	0.1	0.1	0.75	10
6	0.1	0.1	1	10
6	0.1	0.2	0	10
6	0.1	0.2	0.25	10
6	0.1	0.2	0.5	10
6	0.1	0.2	0.75	10
6	0.1	0.2	1	10
6	0.2	0	0	10
6	0.2	0	0.25	10
6	0.2	0	0.5	10
6	0.2	0	0.75	10
6	0.2	0	1	10
6	0.2	0.1	0	10
6	0.2	0.1	0.25	10
6	0.2	0.1	0.5	10
6	0.2	0.1	0.75	10
6	0.2	0.1	1	10
6	0.2	0.2	0	10
6	0.2	0.2	0.25	10
6	0.2	0.2	0.5	10

Continued on next page

Table S1 – continued from previous page

$f(Hz)$	$f_r(Hz)$	$\Delta f(Hz)$	σ_Φ	Number of realization
6	0.2	0.2	0.75	10
6	0.2	0.2	1	10
6	0.3	0	0	10
6	0.3	0	0.25	10
6	0.3	0	0.5	10
6	0.3	0	0.75	10
6	0.3	0	1	10
6	0.3	0.1	0	10
6	0.3	0.1	0.25	10
6	0.3	0.1	0.5	10
6	0.3	0.1	0.75	10
6	0.3	0.1	1	10
6	0.3	0.2	0	10
6	0.3	0.2	0.25	10
6	0.3	0.2	0.5	10
6	0.3	0.2	0.75	10
6	0.3	0.2	1	10
7	0.1	0	0	10
7	0.1	0	0.25	10
7	0.1	0	0.5	10
7	0.1	0	0.75	10
7	0.1	0	1	10
7	0.1	0.1	0	10
7	0.1	0.1	0.25	10
7	0.1	0.1	0.5	10
7	0.1	0.1	0.75	10
7	0.1	0.1	1	10
7	0.1	0.2	0	10
7	0.1	0.2	0.25	10
7	0.1	0.2	0.5	10
7	0.1	0.2	0.75	10
7	0.1	0.2	1	10
7	0.2	0	0	10
7	0.2	0	0.25	10
7	0.2	0	0.5	10
7	0.2	0	0.75	10
7	0.2	0	1	10
7	0.2	0.1	0	10
7	0.2	0.1	0.25	10
7	0.2	0.1	0.5	10
7	0.2	0.1	0.75	10

Continued on next page

Table S1 – continued from previous page

$f(Hz)$	$f_r(Hz)$	$\Delta f(Hz)$	σ_Φ	Number of realization
7	0.2	0.1	1	10
7	0.2	0.2	0	10
7	0.2	0.2	0.25	10
7	0.2	0.2	0.5	10
7	0.2	0.2	0.75	10
7	0.2	0.2	1	10
7	0.3	0	0	10
7	0.3	0	0.25	10
7	0.3	0	0.5	10
7	0.3	0	0.75	10
7	0.3	0	1	10
7	0.3	0.1	0	10
7	0.3	0.1	0.25	10
7	0.3	0.1	0.5	10
7	0.3	0.1	0.75	10
7	0.3	0.1	1	10
7	0.3	0.2	0	10
7	0.3	0.2	0.25	10
7	0.3	0.2	0.5	10
7	0.3	0.2	0.75	10
7	0.3	0.2	1	10
Total number of the simulation: $135 \times 10 = 1350$				
